# Taste Dysfunction and Long COVID-19

**DOI:** 10.3389/fcimb.2021.716563

**Published:** 2021-07-14

**Authors:** Mythily Srinivasan

**Affiliations:** Department of Oral Pathology, Medicine and Radiology, Indiana University School of Dentistry, Indiana University Purdue University at Indianapolis, Indianapolis, IN, United States

**Keywords:** taste buds, epithelial exfoliation, SARS-CoV-2, long COVID-19, dysbiosis

## Introduction

Severe acute respiratory syndrome coronavirus 2 (SARS-CoV-2), the causative agent for the coronavirus disease 2019 (COVID-19), has imposed unprecedented morbidity and mortality worldwide. As of June 2021, globally over 163 million individuals are infected and nearly 3.4 million individuals have died. Emerging concerns include complaints of persistent symptoms for extended periods in recovered individuals. Cellular damage due to disease and/or treatment, prolonged viral shedding, chronic immune inflammatory response, and pro-coagulant state induced by SARS-CoV-2 infection are suggested mechanisms contributing to the symptom sequelae (Estiri et al., 2021; Tran et al., 2021).

## Smell and Taste Disorders in COVID-19

Chemosensory dysfunctions including anosmia, hyposmia, ageusia, and hypogeusia constitute one of the chief symptoms of SARS-CoV2 infection. Meta-analyses suggest that the prevalence of olfactory dysfunction ranged between 41.0–61.0% and that of gustatory dysfunction between 38.2-49.0% in COVID-19. Indeed, self-reported loss of smell and taste has been observed to be more prognostic than other symptoms including fatigue, fever, or cough in predicting symptomatic infection (Agyeman et al., 2020; Mastrangelo et al., 2021). Significantly, loss of taste is consistently reported as a common symptom of long COVID-19, defined as persistence of symptoms four weeks after infection (Biadsee et al., 2021). Following over four-hundred SARS-CoV-2 infected individuals for severity, improvement, and recovery of subjective chemosensory dysfunction for four months, Schwab et al. have reported that the recovery from loss of taste became stagnant after about two months with little improvement subsequently (Schwab et al., 2021). An overview of emerging research on the pathogenesis of long COVID-19 and an opinion about potential mechanisms for gustatory dysfunction is included below.

## Gustation- the Process of Taste Perception

Gustation is an integrated event of multiple physiological processes occurring concurrently through activation of specialized taste, orosensory, and gastrointestinal fibers (Simon et al., 2006). The taste buds, that constitute the peripheral chemosensory units, are distributed in the papillae of the tongue, palate, larynx, and esophagus. Each taste bud consists of 50–100 tightly packed specialized epithelial cells called taste receptor cells that are of three types. Type-I are glial-like cells, type-II cells express G-protein coupled receptors (GPCR) for sweet, bitter, or umami tastes and type-III are presynaptic cells. Clusters of taste receptor cells are chemically and electrically coupled by gap junctions allowing transfer of information intercellularly. The taste buds open on their apical end through a pore filled with microvilli (Simon et al., 2006; Roper, 2013).

The taste buds are innervated by the cranial nerves V, VII, IX and X that transmit information about the chemical nature and quantity of the tastants. Furthermore, these cells are intercalated and surrounded by general sensory thermoreceptors and mechanoreceptors that transduce information about the thermal and physical properties of foods. Collectively, the peripheral gustatory system combines and conveys the multisensory information from foods through multiple neural pathways to the brainstem structures culminating in specific taste perception (Simon et al., 2006; Roper, 2013).

## The “Tongue Film” and Taste Perception

The tastants perfuse through a mucosal film covering the dorsum of the tongue to the apical opening of the taste buds to stimulate the taste receptor cells (Neyraud and Morzel, 2019). This ‘tongue film’ provides unique ecological niche and large surface area for microbial colonization, the metabolization products of which modulate the threshold for specific taste sensitivity (Neyraud and Morzel, 2019; Mantelet et al., 2020). Although widely evaluated in conditions of excess coating, few studies have reported on the composition of microbes in healthy tongue film. Next generation DNA sequencing showed that the tongue film in healthy individuals is rich in bacterial species of the *Fermicutes phylum* that metabolize lactate producing acetate and proprionate (Feng et al., 2018). A high proportion of acetate in the tongue film could increase threshold for sweet perception. Similarly, high concentration of organic acids in the vicinity of the taste receptor cells reduce the sensitivity for fat perception (Neyraud and Morzel, 2019; Gardner et al., 2020).

The taste bud cells undergo continuous renewal with an average turnover rate of 8-12 days and homeostasis is dependent upon a regular supply of properly differentiated taste receptor cells (Roper, 2013). The rapid turnover results in crowding which in turn increases the rate of cell extrusion and apoptosis to achieve epithelial homeostasis (Eisenhoffer et al., 2012). Thus, the epithelial cells in healthy ‘tongue film’ are in different stages of differentiation and include parabasal, intermediate and superficial keratinized cells (Liang et al., 2013). Ultrastructure observations showed that the formation of tongue film is closely related to the rate of multiplication of epithelial cells, quantity of desmosomes and membrane-coating granules (Mantelet et al., 2020).

Interestingly, microbial composition of the dorsum of the tongue and saliva have been observed to be similar, attributed to the presence of exfoliated epithelial cells including the taste bud cells in saliva (Feng et al., 2018). The microbes adhere to the epithelial cells either directly *via* filaments/fimbriae or indirectly through the mucosal film (Kullaa et al., 2014; Neyraud and Morzel, 2019). Further, the saliva in tongue film could also affect taste perception by solubilizing, diluting or otherwise chemically modifying the tastants (Feng et al., 2018).

## Taste Dysfunction-Hypogeusia and Dysgeusia

Taste dysfunction could be a result of local epithelial disorders including damage to the gustatory papillae and taste buds or a result of neuronal disorders such as damage to the peripheral chemosensory units or central lesions (Ambaldhage et al., 2014).

### Role of Tongue Film Microbiota

Considerable evidence suggests that bidirectional mechanisms between the commensal microbiota and the invading virus influence viral infectivity. For example, dysbiosis secondary to the invading virus could increase the prevalence of pathogenic or opportunistic microbes which in turn modulate the innate host responses and induce mediators with either permissive or suppressive effects on viral infection (Dominguez-Diaz et al., 2019; Li et al., 2019). In human immunodeficiency virus (HIV) infection, the commensal (*Veillonella* and *Streptococci*) microbiota was significantly reduced in saliva with concurrent increase in pathogenic bacteria including *Megasphaera, Campylobacter, Veillonella* and *Prevotella species*. In addition, the fungal communities changed significantly with relative abundance of *Epicoccum, Candida* and *Alternaria* and reduced prevalence of *Pichia* species. Interestingly, the decrease in *Pichia* species that normally suppress *Candida* has been related to the increased prevalence of oral candidiasis in HIV infection (Annavajhala et al., 2020). Herpes simplex virus infection has been shown to antagonize oral epithelial cells against *Staphylococcus aureus* adherence but facilitate *Candida albicans* adherence (Plotkin et al., 2016). Following influenza virus infection, the number of pathogenic pathogenic *Pseudomonas* and *Bacillus* was significantly increased and that of non-pathogenic *Prevotella, Veillonella*, and *Neisseria* was decreased in the oropharynx of patients with pneumonia (Leung et al., 2013). In hand foot and mouth disease, the clinical manifestations have been suggested to be due to the combined effects of the causative enterovirus and the induced disruption of the microbiome (Ho et al., 2021).

### Role of Tongue Film Epithelial Cells

Mammalian taste bud cells express several pro-inflammatory cytokines that affect cell renewal, turnover, and function (Wang et al., 2007). While commensal bacteria induce balanced inflammatory responses and maintain host–microbe homeostasis, dysbiosis secondary to viral invasions disrupts the balance and upregulates inflammatory responses (Dominguez-Diaz et al., 2019; Li et al., 2019; Gardner et al., 2020). In herpetic infections, spectral cytopathology showed that the exfoliating oral epithelial cells are morphologically normal but exhibit biochemical composition consistent with degradation of host proteins and synthesis of viral proteins (Papamarkakis et al., 2010). In HIV infection, increased exfoliation of lingual epithelial that exhibit high nuclear/cytoplasmic ratio suggests accelerated turnover or apoptosis that could contribute to the loss of taste (Pompermayer et al., 2011; Dietrich et al., 2012). In influenza virus infection reduced secretion of growth factors that inhibit stem cell activity, mediate inflammation and apoptosis of taste bud cell apoptosis have been suggested as mechanisms for the chronic taste and smell loss (Henkin et al., 2013; Risso et al., 2020). Interestingly, significant proportion of severely diseased SARS-CoV-1 individuals have been shown to present predominantly pale red tongue supporting exfoliation of less differentiated cells (Zou et al., 2003).

## Pathogenesis of Taste Dysfunction in Long COVID-19

The oral epithelial cells including the taste bud cells have been shown to express angiotensin-receptor-2 (ACE2), the entry receptor for viruses of the *Coronaviridae* family including the SARS-COV-2. Emerging evidence also suggest that the CoV-2 potentially uses multiple entry receptors such as the sialic acid receptors and the toll like receptors (TLR) for host cell entry (Vaira et al., 2020; Gadanec et al., 2021). Binding of SARS-CoV-2 to salivary sialic acid could interfere with the glycoproteins mediated transport of tastants and contribute to loss of taste (Milanetti et al., 2021). In-situ models of direct binding of coronavirus spike protein with TLR1, 4 and 6 support the specific roles of these TLRs in CoV-2 entry and COVID-19 (Choudhury and Mukherjee, 2020). Interestingly, taste bud cells express TLRs more abundantly than the non-gustatory lingual epithelium. Specifically, TLRs 2.3 and 4 are highly observed in the gustducin-expressing type II taste bud cells (Wang et al., 2009).

Thus, the expression of multiple entry receptors makes taste bud cells highly susceptible for SARS-CoV-2 infections. Significantly, SARS-CoV-2 viral infection and replication has been shown to occur in human taste bud cells (Doyle et al., 2021). Thus, direct infection of the taste bud cells and consequent inflammation could affect taste perception (Wang et al., 2009; Ambaldhage et al., 2014; Risso et al., 2020). Appropriately, gustatory dysfunctions have been shown to correlate with high serum IL-6, a key cytokine associated with acute and persistent SARS-CoV-2 infection (Cazzolla et al., 2020). Additionally, inflammation could increase epithelial cell exfoliation and constitute potential sources of viral RNA in saliva. Since the viral shedding has been observed for extended period after SARS-CoV-2 infection, it is likely that these epithelial cells could serve as reservoirs (Park et al., 2020; Yang et al., 2020).

Viral invasion also promotes a favorable environment for co-infections that could lead to severe clinical outcomes and mortality (Cox et al., 2020; Ngo and Gewirtz, 2021). In this context, many opportunistic oral pathogens have been observed in the bronchoalveolar lavage fluid supporting a role for oral bacterial co-infections in COVID-19 lung pathology (Bao et al., 2020). Analyzing oropharyngeal swabs from hospitalized COVID-19 patients, Iebba et al. reported that a select panel of oral bacteria and cytokines is predictive of neurological symptoms including hyposmia and dysgeusia in SARS-CoV2 infected individuals. Specifically, the predominance of *Prevotella salivae* and *Veillonella infantium* correlated with the increase in inflammatory cytokines in oral samples in COVID-19 patients. More importantly, the oral bacterial signature and cytokine panel correlated with the serum cytokine profile in hospitalized COVID-19 patients (Iebba et al., 2020; Ma et al., 2021). Interestingly, similar specific oral bacterial preponderance that influenced pneumonia development was previously reported in influenza infections (Gu et al., 2020).

## Opinion and Discussion

**Figure 1:**
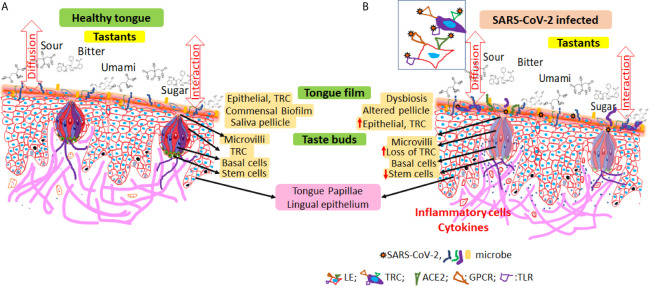
Schematic representation of potential mechanisms for taste dysfunction in long COVID-19. **(A)** The lingual epithelium is covered by a ‘tongue film’ that includes extruded/exfoliated cells, microbiota, and residual saliva. The concentration of microbial metabolization products and the cellular density in the tongue film module taste sensitivity. The tastants diffuse through the tongue film either unaltered or modulated by the microbial metabolization products to reach taste receptor cells through the apical opening of the taste buds. Each taste bud includes tightly packed taste receptor cells, supporting (basal) cells as well as stem cells which replenish the continuously exfoliating taste receptor cells. Commensal microbiota on the dorsum of the tongue form organized consortia largely around a core of keratinized epithelial cells. Dysbiosis secondary to viral invasion disrupts the commensal homeostasis (increase pathogenic or opportunistic microbes) and induce innate inflammatory responses. Persistent irritation induced host responses and increases epithelial proliferation, extrusion, and exfoliation. Pressure on replenishment for taste receptor cells places increased demand on stem cells and thereby compromises taste bud homeostasis, which in turn affects taste perception. **(B)** Inset shows oral epithelial cells expressing multiple entry receptors for SARS-CoV-2. LE, lingual epithelium; TRC, taste receptor cells; ACE-2, angiotensin converting enzyme-2; TLR, toll like receptor; GPCR, G-protein coupled receptor.

Prolonged dysgeusia and viral shedding suggest anatomical reservoirs for SARS-CoV2 that act as a source for active or latent taste dysfunction in long COVID-19. Here, we propose that altered epithelial homeostasis secondary to viral infection induced dysbiosis and chronic inflammation characterized by increased exfoliation and reduced taste receptor potentially contribute to the persistent dysgeusia in long COVID-19 (**Figure 1**). Viral persistence in the tongue epithelial cells including the taste receptor cells for extended periods after infection could modulate the host responses either by itself or by disrupting the commensal microbiota. While the initial epithelial cell and innate immune responses may prevent or suppress viral invasion, prolonged perturbations of the commensal microbiota will likely precipitate exaggerated inflammatory responses (Li et al., 2019). Mucosal inflammation increases the epithelial cell exfoliation and constitute potential sources of viral shedding in saliva (Herrmann et al., 2002; Groeger and Meyle, 2019). The lag in replenishment of lost cells together with the reduced stem cell turnover could result in fewer taste receptor cells potentially leading to the persistent taste dysfunction. It will be interesting to investigate whether salivary epithelial cell analyses could reveal specific markers of dysgeusia in individuals with long COVID-19.

## Author Contributions

The author confirms being the sole contributor of this work and has approved it for publication.

## Funding

The work was supported by pilot grant from Regenstrief Institute, Indiana University Purdue University at Indianapolis.

## Conflict of Interest

The author declares that the research was conducted in the absence of any commercial or financial relationships that could be construed as a potential conflict of interest.
